# Supplementary material for: A local-global transformer-based model for person re-identification
Source: PLoS One. 2025 Nov 13;20(11):e0335848. doi: 10.1371/journal.pone.0335848 (PMC12614614; doi:10.1371/journal.pone.0335848)
Supplement: Attachment — Submitted filename: Reviewer Attachment PONE-D-25-21383.docx [file pone.0335848.s001.docx]

**Manuscript Number:** PONE-D-25-21383

**Manuscript Title:** A local-global Transformer-based model for Person Re-Identification

I would like to offer several recommendations that the authors might consider beneficial in the process of revising their manuscript:

- It is needed to provide the novelty of this work

- It is suggested to conduct statistical tests for the proposed system. For example, see Section 4.5 in paper [a] and the author could cite this paper for reference.

[a] Biswas, S., Mostafiz, R., Uddin, M.S. and Paul, B.K., 2024. XAI-FusionNet: Diabetic foot ulcer detection based on multi-scale feature fusion with explainable artificial intelligence. Heliyon, 10(10), p.e31228.

- please site the following papers

[1] https://doi.org/10.1016/j.ibmed.2023.100128

[2] https://doi.org/10.1007/s44174-024-00165-5

[3] https://doi.org/10.1007/s10772-024-10152-2
