# Supplementary material for: A local-global transformer-based model for person re-identification
Source: PLoS One. 2025 Nov 13;20(11):e0335848. doi: 10.1371/journal.pone.0335848 (PMC12614614; doi:10.1371/journal.pone.0335848)
Supplement: Attachment — Submitted filename: Response_to_Reviewers_auresp_2.docx [file pone.0335848.s003.docx]

Response to Reviewers for Manuscript ID: PONE-D-25-21383

Dear Editors and Reviewers:

We sincerely appreciate the valuable feedback provided by the Editor and reviewers on our manuscript entitled “A local-global Transformer-based model for Person Re-Identification.” These suggestions have been instrumental in further refining our paper and have provided constructive direction for our ongoing research. We have thoroughly addressed all the feedback and implemented corresponding revisions in the manuscript, with the hope that these adjustments meet your expectations.

Revised portion are marked in yellow in the paper. The main corrections in the paper and the responds to the reviewer’s comments are as flowing:

We ensure that we refer to Table 1,2,3 and 4 in our article.

We sincerely appreciate the Editors’ time and constructive comments, which have significantly improved the quality of our manuscript. We believe the paper now meets the journal’s standards and hope it is deemed suitable for publication.

The source code will be made publicly available upon acceptance (GitHub link: https://github.com/xukeeeee/A-local-global-Transformer-based-model-for-Person-Re-Identification).
